# Differential drought tolerance in tree populations from contrasting elevations

**DOI:** 10.1093/aobpla/plu069

**Published:** 2014-11-10

**Authors:** Fei Ma, Ting Ting Xu, Ming Fei Ji, Chang Ming Zhao

**Affiliations:** 1 New Technology Application, Research and Development Center, Ningxia University, Yinchuan 750021, PR China; 2 School of Life Science, Ningxia University, Yinchuan 750021, PR China; 3 State Key Laboratory of Grassland Agro-Ecosystem, School of Life Sciences, Lanzhou University, Lanzhou 730000, PR China

**Keywords:** Carbon isotope composition, drought tolerance, growth, leaf gas exchange, *Pinus tabuliformis*, water-use efficiency.

## Abstract

To predict the ecological consequences of climate change for a widely distributed tree species, it is essential to develop a deep understanding of the ecophysiological responses of populations from contrasting climates to varied soil water availabilities. In the present study, we focused on *Pinus tabuliformis*, one of the most economically and ecologically important tree species in China. In a greenhouse experiment, we exposed trees from high-elevation (HP) and low-elevation (LP) populations to low (80 % of field capacity, FC), mild (60 % FC), moderate (40 % FC) and severe (20 % FC) water stresses. Leaf gas exchange, biomass production and allocation, as well as water-use efficiency, were measured during the experiment. Increasing soil water stress clearly decreased the relative growth rate (RGR), total dry mass (TDM), light-saturated photosynthetic rate (*A*_sat_), stomatal conductance (*g*_s_), total water use (TWU) and whole-plant water-use efficiency (WUE_WP_). In contrast, intrinsic water-use efficiency (WUE_i_) and carbon isotope composition (δ^13^C) both increased significantly with increasing soil water stress for both populations. Only in the LP did the root/shoot ratio (R/S ratio) significantly increase when the water stress increased. A strong positive correlation between *A*_sat_ and *g*_s_ coupled with a reduced intercellular CO_2_ concentration (*C*_i_) probably suggested that stomatal limitations were the main cause of the decreased *A*_sat_. However, all the measured variables from the HP were affected less by drought compared with those of the LP, and most aspects of the HP were canalized against drought stress, which was reflected by the relatively higher RGR, TDM and WUE_WP_. Overall, the results suggest that the two populations responded differentially to drought stress with the HP showing higher drought tolerance than the LP, which was reflected by its faster seedling growth rate and more efficient water use under drought conditions.

## Abstract (precis)

To investigate the differential drought tolerance between populations of *Pinus tabuliformis* from high-elevation (HP) and low-elevation (LP), seedlings of these two populations were subjected to a gradual depletion of soil water availability with a series of traits related to growth and water use efficiency being measured. From our results, we found that all the measured variables from the HP were affected less by drought compared to those of the LP, and most aspects of the HP were canalized against drought stress. We thus drew a conclusion that the two populations responded differentially to drought stress with the HP showing higher drought tolerance than the LP.

## Introduction

Water availability is a crucial factor that limits the growth, development and distribution of all plants (Chaves *et al.* 2003; Ordoñez *et al.* 2009; Wu *et al.* 2010), and its importance will only become more pronounced in the future due to human-caused climate change resulting in more frequent and severe drought events (IPCC 2007). Therefore, to predict the ecological consequences of climate change on the widely distributed tree species, detailed knowledge on their ability to cope with varied water availability is needed within and among populations.

Low water availability (drought) affects the performance of plants by affecting their morphological, physiological and biochemical, as well as transcriptomic and proteomic processes (Anyia and Herzog 2004; González-Rodríguez *et al.* 2005; Dias *et al.* 2007; Foito *et al.* 2009; Gao *et al.* 2009; Ma *et al.* 2010; Tomlinson *et al.* 2012). A gradual depletion of the soil water leads to the stomatal (*g*_s_) and mesophyll (*g*_m_) conductance being reduced, and this is believed to be the primary drought stress response (Flexas *et al.* 2008; Chaves *et al.* 2009; Galmés *et al.* 2011; Warren *et al.* 2011), which causes the water loss to be reduced, but this also results in the rate of photosynthesis being reduced due to reduced CO_2_ in chloroplasts (Flexas *et al.* 2008). Photosynthesis can be further limited by metabolic impairment due to increasing drought stress (Flexas *et al.* 2008). Drought can also lead to reduced growth and biomass production, while also altering the allocation pattern of biomass (Erice *et al.* 2010).

Water-use efficiency (WUE) is one of the most important indicators for evaluating the tolerance of plants to water stress (Kozlowski and Pallardy 1997). At the leaf level, WUE can be defined as the ratio of the net photosynthetic rate (*A*_N_) to *g*_s_ (WUE_i_, intrinsic water-use efficiency), and an integrated measurement of WUE_i_ can be reliably assessed by the carbon isotope composition (δ^13^C, a measure of the ^13^C/^12^C ratio in plant tissues compared with air) as it has a linear relationship with the intercellular to ambient CO_2_ ratio (*C*_i_/*C*_a_) (Farquhar *et al.* 1989; Brodribb and Hill 1998). Factors that affect *g*_s_ and *g*_m_ can thus influence *C*_i_ and subsequently the relationship between WUE_i_ and δ^13^C (Flexas *et al.* 2008; Seibt *et al.* 2008; Fleck *et al.* 2010). Water-use efficiency at the whole-plant level, defined as the ratio of actual dry matter production to water consumption (WUE_wp_), represents a large spatial (whole plant) and temporal scale (whole growth period) water use that is closely associated with the physiological processes of plants, such as photosynthesis, respiration and transpiration (Flexas *et al.* 2010). If a plant has a greater WUE, it is expected to be able to survive environments that are more arid better than a plant with a lower WUE (Jones 1992; Ares *et al.* 2000; Franco *et al.* 2005).

*Pinus tabuliformis* is an endemic pine species from China which is one of the most economically and ecologically important tree species in the northern part of the country and covers a total area of 228.10 × 10^4^ ha. Particularly in arid and semi-arid areas, it plays an important role in reforestation (Zhao and Zhou 2005). Due to its wide geographical distribution and long life span, populations *of P. tabuliformis* are exposed to a wide variety of drought stresses that has likely led to the adaptation of natural populations to locally distinct environments. Therefore, determining how populations have adapted to varied soil water conditions will enable a greater understanding of past differentiation while also enabling better forest management and restoration in the future (Yong *et al.* 2000). In the present study, two populations of *P. tabuliformis* from contrasting elevations were selected and subjected to a gradient of soil water contents, due to the species having occurred over a wide range of elevations from 100 to 2800 m above sea level (Chen *et al.* 2008). Relative to populations growing at lower elevations, tree populations from higher elevations generally exhibit reduced growth, smaller and thicker leaves, higher leaf nutrient content per unit area, higher fine root production and higher allocation of biomass to roots (Oleksyn *et al.* 1998; Körner 1999; Zhao *et al.* 2008; Bresson *et al.* 2011; Petit *et al.* 2011). The differentiation in these physiological and morphological traits has been thought to be an adaptation to enhance photosynthesis and water-use efficiency while increasing the resistance to the limited water availability (Oleksyn *et al.* 1998; Körner 1999; Bresson *et al.* 2011). Therefore, we expected that the two populations would show differential responses to varied soil water availabilities, with the population from the high elevation (HP) having a higher drought tolerance than the low-elevation population (LP), which would result in a higher growth rate, biomass production and water-use efficiency under limited water conditions.

## Methods

### Plant material and experimental design

Seeds of *P. tabuliformis* for use in the present study were collected from two locations: Xiahe (35°33.85′E, 102°13.60′N, 2810 m Alt.; HP) and Zhengning (35°31.18′E, 108°29.51′N, 1444 m Alt.; LP). The corresponding mean annual rainfall values in the two areas are 516 and 623 mm, while the mean annual temperatures (MATs) are 3.6 and 9.6 °C, respectively. These seeds were germinated and grown indoors for 1 year in a tree nursery, and 112 seedlings of each population with no statistical differences in height and size were transferred to Yuzhong, Gansu Province (35°56.61′N; 104°09.07′E; 1750 m Alt.), and immediately replanted into 6-L plastic pots (28 pots, four seedlings per pot) filled with the same weight of a homogeneous mixture (peat and perlite, 1 : 1 by volume). Another 12 pots were prepared in the same way but without seedlings and these were used to determine the evaporation of water from the soil. The soil surface in all the pots was covered with a small quantity (c. 2 cm) of perlite to minimize evaporation. The maximum field capacity (FC) for watering the pots was determined gravimetrically according to Shou *et al.* (2004) with some modifications. All pots were periodically watered to FC for 2 months after repotting to allow the seedlings to become established. The seedlings were grown for the rest of the study in a canopied and naturally lit glasshouse, the roof of which was closed at night and on rainy days, but opened during any day it was not raining. The sides of the glasshouse were always open for aeration during the whole experiment, so that the temperature inside the glasshouse was closely linked to the outside ambient temperature.

For each population, 20 pots were selected and divided into four lots of five pots each (low, mild, moderate and severe water stress treatments). The remaining pots were used to determine the initial biomass. Water stress treatments were achieved by watering to 80 % of maximum FC, 60 % FC, 40 % FC and 20 % FC. All water stress treatments reached the target FC in 7 days from the beginning of the experiment. Soil water content was maintained by weighing the pots every 2 days, recording the water loss and re-watering to the designated water level immediately. The soil water contents before and after watering were maintained at 54–60, 45–50, 34–40 and 22–25 % for the treatments, respectively. The experiment lasted for 134 days from July to November, and during the whole experiment no fertilizer was added at any point and no plants died.

### Leaf gas exchange

On 3 sunny days (15 August, 15 September and 15 October) during the experiment, the light-saturated photosynthetic rate (*A*_sat_), stomatal conductance (*g*_s_) and intercellular CO_2_ concentration (*C*_i_) were measured on sun-adapted needles using an LI-COR 6400 infrared gas-analyzer (IRGA, LI-COR, Lincoln, NE, USA). The light level was maintained at 1500 μmol m^−2^ s^−1^ using an LI-6400-02B LED light source (10 % blue light) and the external CO_2_ concentration was maintained at 370 μmol mol^−1^ using a CO_2_ injector (LI-6400-01). The ambient and internal temperatures and vapour pressure deficits were 31.03 ± 1.18 °C, 3.18 ± 0.53 kPa and 31.50 ± 0.11 °C, 3.35 ± 0.20 kPa on 15 August; 27.00 ± 1.08 °C, 2.52 ± 0.17 kPa and 27.67 ± 0.35 °C, 2.83 ± 0.32 kPa on 15 September and 21.30 ± 0.83 °C, 2.20 ± 0.17 kPa and 21.89 ± 0.22 °C, 2.31 ± 0.14 kPa on 15 October, respectively. At least four replicates for each treatment per population were measured and measurements of two individual seedlings in one pot were considered as one replicate. Needles were marked and cut after the last measurement for area determination using an LI-COR-3000A planimeter (LI-COR, Lincoln, NE, USA). The WUE_i_ was defined as the ratio of *A*_sat_ to *g*_s_. The mean values of *A*_sat_, *g*_s_, *C*_i_ and WUE_i_ measured on 3 days are presented in this paper.

### Growth and water use

Due to possible within pot effects, such as competition for resources, each pot was considered to be a single replicate with the four seedlings’ measurements being combined for determining the growth and water use. To estimate the biomass production during the experiment, three pots (12 seedlings) from each population at the beginning of the experiment (*t*_1_) and four pots (16 seedlings) at the end of the experiment (*t*_2_) were harvested. From each pot, the four seedlings were bulked together and divided into three parts: leaves, stems and roots. The three biomass parts were dried for 48 h at 80 °C in an oven, weighted and then the weights were divided by four to determine per plant values from the per pot values. The relative growth rate (RGR) was calculated using the following formula: RGR = (ln*W*_2_ − ln*W*_1_)/(*t*_2_ − *t*_1_), where *W*_1_ and *W*_2_ are the dry weights per plant at Day *t*_1_ and Day *t*_2_. The root/shoot (R/S) ratio was also calculated. The WUE at the whole-plant level was calculated as WUE_WP_ per plant = (*W*_2_ − *W*_1_)/*T*, where *T* is the total transpired water use per plant (TWU) between *t*_1_ and *t*_2_.

### Carbon isotope composition

The oven-dried needle samples were finely ground with a Tissuelyzer (Retsch, Haan, Germany), and the carbon isotope composition of the needles (δ^13^C) was determined by combusting the samples in an elemental analyser EA1108 (Carlo Erba, Milano, Italy) coupled to a Finnigan Delta Plus isotope mass spectrometer (Thermo Finnigan MAT GmbH, Bremen, Germany) at the Key Laboratory of Western China's Environmental Systems (Ministry of Education), Lanzhou University. The carbon isotope composition was calculated relative to the Pee Dee Belemnite (PDB) standard as the ratio (‰): δ^13^C = [(*R*_sample_/*R*_standard_) − 1] × 1000, where *R*_sample_ and *R*_standard_ are the ratios of ^13^C/^12^C in the sample and the standard, respectively.

### Statistical analyses

The variables including LDM, SDM, RDM, TDM, RGR, R/*S* ratio, TWU, WUE_wp_ and δ^13^C were analysed using the general linear model (Proc GLM) to test the effect of the populations, water treatments and their interactions. Leaf gas exchange parameters, including *A*_sat_, *g*_s_, *C*_i_ and WUE_i_, were analysed by the GLM with the measurement time as a covariate. When the differences were significant, a multiple comparison of means (post-hoc Tukey's honestly significant difference test) was carried out. Before the statistical tests were performed using the SPSS software package (SPSS, Inc., Chicago, IL, USA), the homogeneity of the data was determined.

## Results

### Plant growth, biomass production and allocation

**Figure 1.**
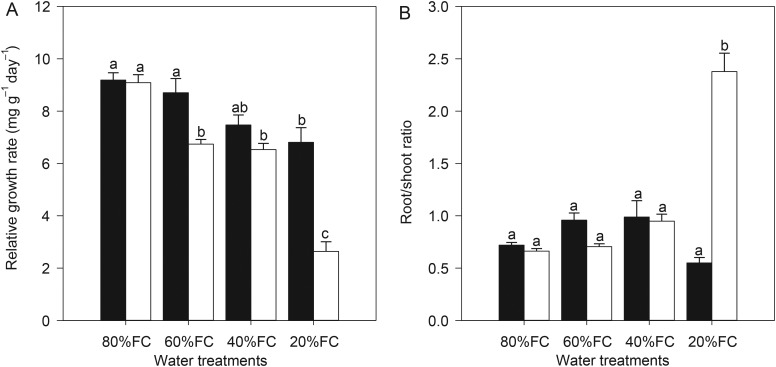
Relative growth rate and R/S ratio in two populations of *Pinus tabuliformis* from a HP (black bars) and a LP (white bars) under various soil water conditions (80 % of maximal FC, 60 % FC, 40 % FC and 20 % FC). Scale bars represent mean ± SE. The letters indicate statistical differences (*P* < 0.05) for the water treatments, populations and the interactions between them.

As the available soil water decreased, the dry mass of leaves (LDM), stems (SDM) and roots (RDM) decreased in both populations, which leads to a decrease in total dry mass (TDM); RGR was also reduced (Table 1, Fig. 1). Compared with the seedlings exposed to the low water stress, the severe water stress resulted in a significant decrease in the TDM by 38 and 82 % and the RGR by 26 and 71 % for the HP and LP, respectively (Table 1, Fig. 1). The values of the RGR and TDM were higher in the HP than those in the LP across mild, moderate and severe stress treatments (Table 1, Fig. 1). The dry mass allocation differed significantly between the HP and LP as the water stress increased (Fig. 1). The R/S ratio increased by a factor of 1.54 for the LP from low to severe water stress, but there were only slight changes between the low water stress and the other three treatments in the HP (Fig. 1). The interactions between the populations and treatments for these variables were also highly significant (Table 2).

**Table 1.** Growth, biomass production and allocation as well as water use of *Pinus tabuliaeformis* from a HP and a LP under various soil water conditions (80 % of maximal FC, 60 % FC, 40 % FC and 20 % FC). Each point represents mean ± SE. The letters indicate statistical differences (*P* < 0.05) for the water treatments, populations and the interactions between them.

	Water treatments			
	80 % FC	60 % FC	40 % FC	20 % FC
Leaf dry mass (LDM) (g)				
HP	2.00 ± 0.04^a^	1.81 ± 0.26^ab^	1.20 ± 0.12^b^	1.67 ± 0.28^ab^
LP	2.02 ± 0.11^a^	1.24 ± 0.11^b^	1.04 ± 0.18^b^	0.13 ± 0.03^c^
Stem dry mass (SDM) (g)				
HP	1.23 ± 0.09^ab^	0.83 ± 0.11^bc^	0.82 ± 0.16^bc^	0.59 ± 0.07^cd^
LP	1.44 ± 0.14^a^	0.84 ± 0.02^bc^	0.70 ± 0.03^c^	0.18 ± 0.04^d^
Root dry mass (RDM) (g)				
HP	2.33 ± 0.17^ab^	2.46 ± 0.17^ab^	1.93 ± 0.15^bc^	1.19 ± 0.08^de^
LP	2.29 ± 0.14^ab^	1.46 ± 0.04^cd^	1.64 ± 0.03^cd^	0.72 ± 0.10^e^
TDM (g)				
HP	5.55 ± 0.29^a^	5.10 ± 0.54^ab^	3.95 ± 0.33^bc^	3.45 ± 0.42^c^
LP	5.75 ± 0.34^a^	3.54 ± 0.14^c^	3.38 ± 0.18^c^	1.04 ± 0.17^d^
TWU (g)				
HP	1.34 ± 0.06^a^	1.53 ± 0.14^ac^	1.63 ± 0.08^ac^	0.85 ± 0.04^bd^
LP	1.79 ± 0.05^c^	1.25 ± 0.08^ab^	1.57 ± 0.04^ac^	0.85 ± 0.12^d^
WUE_wp_ (g kg^−1^)				
HP	4.13 ± 0.04^a^	3.33 ± 0.04^bc^	2.42 ± 0.09^cd^	4.00 ± 0.33^ab^
LP	3.21 ± 0.12^bc^	2.86 ± 0.29^cd^	2.15 ± 0.06^d^	1.21 ± 0.03^e^

**Table 2.** Comparison of all variables measured in the experiment. The *P-*values are presented for the watering treatments, populations and their interactions. *P < 0.05; **P < 0.01; ***P < 0.001.

Variables	Treatment (*T*)	Population (*P*)	*T* × *P*
LDM	20.52***	26.86***	10.45***
SDM	32.73***	1.36	3.67*
RDM	44.33***	27.00***	5.55**
TDM	38.92***	22.84***	6.32**
RGR	49.63***	47.07***	11.31***
R/S ratio	27.68***	33.12***	57.88***
*A*_sat_	101.09***	69.00*	2.57***
*g*_s_	102.77***	131.99***	13.88***
*C*_i_	103.80***	253.01***	12.06***
WUE_i_	37.55**	146.34***	13.49***
TWU	36.10***	0.194	6.97**
WUE_L_	35.67***	119.75***	12.23***
δ^13^C	39.11***	25.84***	7.51**

### Leaf gas exchange

**Figure 2.**
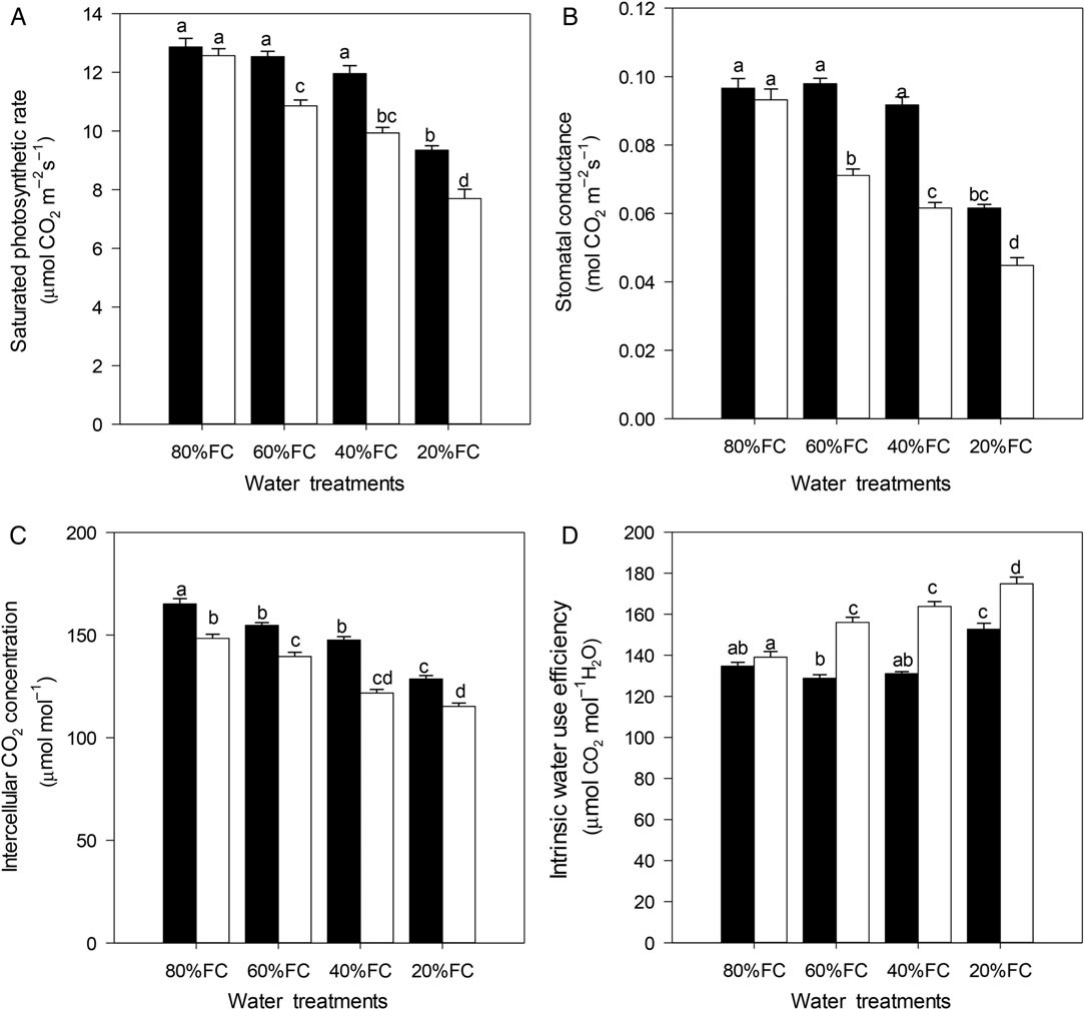
Means of light-saturated photosynthetic rate (*A*_sat_), stomatal conductance (*g*_s_), intercellular CO_2_ concentration (*C*_i_) and intrinsic water-use efficiency (WUE_i_) measured on 3 days (15 August, 15 September and 15 October) in two populations of *Pinus tabuliformis* from a HP (black bars) and a LP (white bars) under various soil water conditions (80 % of maximal FC, 60 % FC, 40 % FC and 20 % FC). Each bar represents mean ± SE. The letters indicate statistical differences (*P* < 0.05) for the water treatments, populations and the interactions between them.

**Figure 3.**
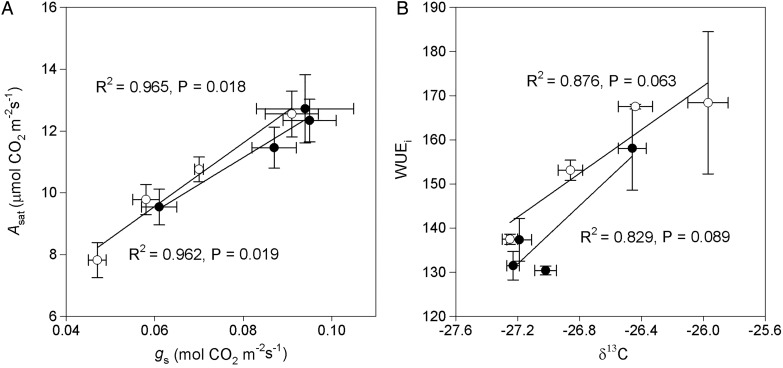
Relationships between light-saturated photosynthetic rate (*A*_sat_) and stomatal conductance (*g*_s_) as well as between WUE_i_ and carbon isotope composition (δ^13^C) in the two populations of *Pinus tabuliformis* from a HP (filled circles) and a LP (empty circles) across water treatments. The coefficient of determination (*R*^2^) and significance are shown for each regression.

An increased water stress resulted in a significantly reduced *A*_sat_, *g*_s_ and *C*_i_ in both populations (Fig. 2, Table 2). However, the reductions in *A*_sat_, *g*_s_ and *C*_i_ followed different patterns for the different populations investigated. Much of the decline of *A*_sat_, *g*_s_ and *C*_i_ occurred under severe water stress in the HP, but for the LP the declines were more gradual (Fig. 2). Severe water stress decreased the *A*_sat_ by 27 and 39 %, *g*_s_ by 36 and 52 % and *C*_i_ by 22 and 27 % for the HP and LP, respectively. The greater decreases in *g*_s_ compared with *A*_sat_ led to a 15 and 22 % increase in the WUE_i_ for the HP and the LP, respectively (Fig. 2). The effects of the populations, treatments and their interactions were also significant on those variables (Table 2). In addition, for both populations, there were strong positive correlations for the *A*_sat_ and *g*_s_ variables (Fig. 3).

### Water-use traits

**Figure 4.**
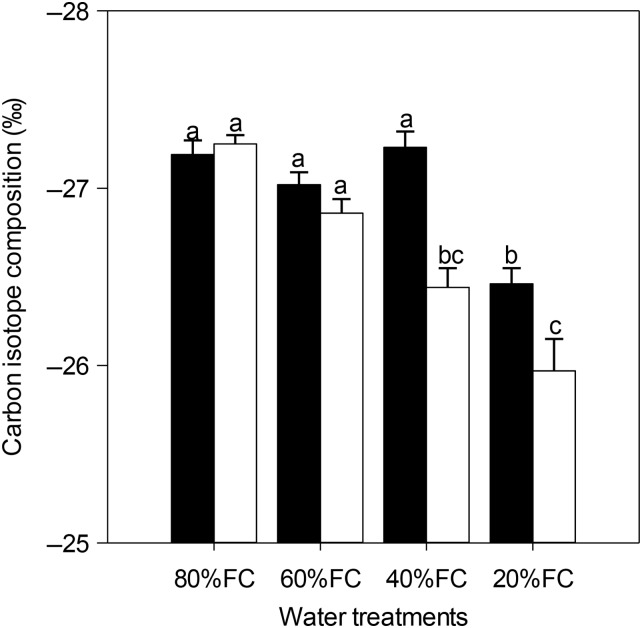
Carbon isotope composition (δ^13^C) in the two populations of *Pinus tabuliformis* from a HP (black bars) and a LP (white bars) under various soil water conditions (80 % of maximal FC, 60 % FC, 40 % FC and 20 % FC). Each point represents mean ± SE. The letters indicate statistical differences (*P* < 0.05) for the water treatments, populations and the interactions between them.

TWU and WUE_WP_ both decreased significantly with decreasing soil water content (Table 1). From the low to moderate stress, a decline in WUE_WP_ was observed in both populations. Severe water stress saw a further decrease in the LP but an increase in the HP. However, the HP exhibited a higher WUE_WP_ than the LP in all the water level treatments and significant differences were observed in the low and severe stress treatments (Table 1). The δ^13^C gradually increased as the water stress increased in the LP, while only the severe water stress induced an increase in δ^13^C for the HP (Fig. 4). The interactions between the populations and treatments for these three variables were also highly significant (Table 2).

## Discussion

Water availability as a growth-limiting factor was demonstrated in the present study, as it caused significant reductions in RGR, TDM, LDM, SDM and RDM in both populations (Table 1, Fig. 1). Comparatively, the HP showed a higher RGR and TDM from the mild to severe water stress treatments than the LP, and the differences were highly significant (Table 1, Fig. 1). These results support the previously published work that various growth responses within and between species were due to drought stress (Bacelar *et al.* 2007; Bruschi 2010; Ma *et al.* 2010). Research has also revealed that plants with higher drought tolerance exhibit less growth inhibition and had relatively higher growth and biomass production than drought-sensitive ones (Loggini *et al.* 1999; Türkan *et al.* 2005). Therefore, these results suggested a higher capacity for the HP than for the LP to sustain growth and production under water-limited conditions.

Drought affects plant growth by influencing the leaf gas exchange rates (Zhang and Marshall 1994; Bacelar *et al.* 2007; Ma *et al.* 2010; Sapeta *et al.* 2013). A reduction in *g*_s_ and *g*_m_ as well as metabolic impairment are considered to be the main causes of the depression of photosynthesis in the face of drought stress (Flexas *et al.* 2008). Accordingly, *g*_s_ and *A*_sat_ of the two populations significantly decreased after exposure to drought stress, and *A*_sat_ was strongly positively correlated with *g*_s_ (Fig. 2). From this it was possible to surmise that stomatal closure caused by drought stress resulted in the *A*_sat_ being reduced under drought conditions (Fig. 3), and the *C*_i_ in both populations being reduced at the same time supports this conclusion (Michelozzi *et al.* 2011). However, compared with the gradual decrease of *g*_s_ and *A*_sat_ in the LP, only severe water stress induced significant reductions in those two parameters in the HP. Even under extreme water stress conditions, the HP had higher *g*_s_ and *A*_sat_ values than the LP (Fig. 2). These results indicated that the leaf gas exchange in the two populations responded differently to the drought conditions, and that the apparent ability of the HP to maintain higher photosynthetic rates may allow it to grow more rapidly under water-limited conditions. This conclusion is supported by the above results that the HP exhibited a higher growth rate and biomass production than the LP under water-limited conditions (Table 1, Fig. 1).

The WUE_i_ and δ^13^C significantly increased in both populations with decreasing water availability, and the WUE_i_ was positively correlated with δ^13^C (Fig. 3), which was similar to the results of previous studies (Farquhar *et al.* 1989; Jones 1993; Zhang and Marshall 1994). The WUE_i_ and δ^13^C of the LP gradually increased from the low to severe water stresses, whereas these two parameters for the HP only showed significant increases under severe stress treatment (Table 2). The higher WUE_i_ and δ^13^C values in the LP than in the HP under mild, moderate and severe water stress treatments were mainly due to the relatively small changes of *A*_sat_ and *g*_s_ in the HP under drought conditions (Table 2). These findings support the hypothesis that populations will be less plastic if they come from an environment that is dry (Volis *et al.* 2002; Heschel *et al.* 2004). Aranda *et al.* (2010) also reported lower plasticity to environmental changes in the HP than in the LP.

With respect to the WUE at the whole-plant level, the WUE_wp_ showed an opposite trend to the WUE_i_ and δ^13^C, with both populations recording a significant drop between the low and moderate stress treatments, and a further significant drop between the moderate and severe stress treatments for the LP (Table 1). These findings confirmed previous observations by Tomás *et al.* (2014) and Flexas *et al.* (2010) that there are large discrepancies when scaling-up WUE measurements from the leaf to the whole-plant level. Several structural and physiological processes, such as canopy structure, transpiration by plant organs other than leaves, respiration by leaf during the night and by stem and root during the whole day, will lead to a decrease in the WUE_wp_, but not influence the leaf-level estimates. However, the HP showed a significantly higher WUE_WP_ than the LP in all water treatments (Table 1), which indicates a higher potential to survive water-limited conditions by efficient water use (Jones 1992).

It is widely accepted that a reduced water supply will result in an increased partitioning of biomass in favour of root growth (Fernández and Reynolds 2000; Khurana and Singh 2004; Nagakura *et al.* 2004), but not all studies have found this (Osório *et al.* 1998; Tomlinson *et al.* 2012). Curiously, in the current study, an increase in the R/S ratio was evident in the LP, whereas in the HP there was no detectable change, which indicates that a loss of plasticity for this character might have been an advantage for existence at higher elevations (Sobrado and Turner 1986; Aranda *et al.* 2010).

## Conclusions

This study indicated that increasing water stress had a significant effect on leaf gas exchange, biomass production and allocation, carbon isotope composition and water-use efficiency in both HP and LP. However, the two populations differed significantly in their responses to drought stress: the HP appeared to be less affected by water stress than the LP as far as the examined variables were concerned, as well as the exhibited TDM, RGR and WUE_L_ in the stress treatments. The results supported the hypothesis that there would be different drought tolerance levels in the two populations with the HP having a greater tolerance.

## Sources of Funding

This study was supported by grants from the National Natural Science Foundation of China (Nos 31260166, 31170571 and 31360185).

## Contributions by the Authors

The research design and preparation of the manuscript are credited to F.M. T.T.X. contributed to data collection and analysis. M.F.J. mainly contributed to the seedling cultivation. C.M.Z. contributed to conception of the study and suggestions for writing the manuscript.

## Conflicts of Interest Statement

None declared.
